# A Novel Homozygote Pathogenic Variant in the DIAPH1 Gene Associated With Seizures, Cortical Blindness, and Microcephaly Syndrome (SCBMS): Report of a Family and Literature Review

**DOI:** 10.1002/mgg3.70031

**Published:** 2024-11-23

**Authors:** Emran Esmaeilzadeh, Sajjad Biglari, Meysam Mosallaei, Hamid Reza Khorram Khorshid, Hassan Vahidnezhad, Mohammad Amin Tabatabaiefar

**Affiliations:** ^1^ Fetal Health Research Center Hope Generation Foundation Tehran Iran; ^2^ Department of Genetics and Molecular Biology, School of Medicine Isfahan University of Medical Sciences Isfahan Iran; ^3^ Center for Applied Genomics Children's Hospital of Philadelphia Philadelphia Pennsylvania USA; ^4^ Department of Pediatrics, Perelman School of Medicine University of Pennsylvania Philadelphia Pennsylvania USA; ^5^ Genetics Research Center University of Social Welfare and Rehabilitation Science Tehran Iran; ^6^ Division of Human Genetics Children's Hospital of Philadelphia Philadelphia Pennsylvania USA

**Keywords:** *DIAPH1*, exome sequencing, microcephaly, SCBMS

## Abstract

**Objective:**

Mammalian Diaphanous‐Related Formin (mDia1), which is encoded by the *DIAPH1* gene, serves as essential for the regulation of cell morphology and cytoskeletal organization. The role of *DIAPH1* in brain development has been extensively established. This study aims to evaluate the clinical, neuroradiological, and genetic characteristics of patients with *DIAPH1*‐related disease and determine probable genotype–phenotype relationships.

**Methods:**

In the current study, exome sequencing was performed to identify the genetic basis of the clinical presentation in an Iranian 7‐year‐old boy. Validation of the detected variant was done by Sanger sequencing. Furthermore, we performed a comprehensive review of the literature.

**Results:**

Here, we detected a novel homozygous c.1285C> T (p.Gln429*) pathogenic variant in the patient. In silico analysis with prediction software tools identified this variant as a probable source of damage. Twenty cases from seven studies were found after a review of the literature. The patients' main symptoms were a developmental delay, microcephaly, and seizures. The mean age of onset for patients in the group of 20 patients with a known age of onset was 2.3 months (SD = 1.6). Of the variants identified, c.2769del, c.684+1G>A, and c.2332C> T were identified in 72% of the patients.

**Conclusion:**

Considering the variant's position in the gene and the encoding protein, a pathogenic effect is predicted for the variant. So, the patient's clinical manifestation is probably caused by this pathogenic variant. Moreover, by studying clinical manifestations in all molecularly confirmed reported cases, provided a comprehensive overview of clinical presentation, and attempted to find a genotype–phenotype correlation.

## Graphical abstract

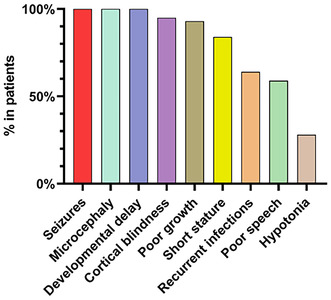

## 1 Introduction

Primary microcephaly (PM) is a congenital neurodevelopmental disorder characterized by a small brain and a head size at least 3 standard deviations (SD) below the mean of age, sex, and ethnicity‐matched controls, as defined by the measurement of occipitalfrontal circumference (OFC) (Jayaraman, Bae, and Walsh 2018). The prevalence of microcephaly ranges between 1.5 and 8.7 per 10,000 births in Europe and the United States, respectively (Morris et al. 2016). PM can have different genetic etiologies and manifests either as a feature in a variety of syndromes or as an isolated condition. The subgroup of nonsyndromic PM is termed “microcephaly primary hereditary” (MCPH) (Jayaraman, Bae, and Walsh 2018). The underlying genetic defects of microcephaly are highly heterogeneous, but with the advent of next‐generation sequencing (NGS) technologies, exact molecular diagnoses are becoming more achievable (Woods and Parker 2013). To date, pathogenic variants in the *DIAPH1* gene have been identified in two conditions. Dominant gain‐of‐function *DIAPH1* variants cause sensorineural deafness and macrothrombocytopenia (DFNA1) (Stritt et al. 2016; Ueyama et al. 2016), while homozygous *DIAPH1* loss of function leads to seizures, cortical blindness, and microcephaly syndrome (SCBMS) (#MIM: 616632) (Al‐Maawali et al. 2016; Ganaha et al. 2017; Kaustio et al. 2021). The *DIAPH1* gene encodes the mammalian Diaphanous‐related formin (mDia1), which acts downstream of Rho GTPases. It has been speculated that the *DIAPH1* gene has an essential role in the regulation of actin polymerization and microtubule stabilization (Ercan‐Sencicek et al. 2015; Esmaeilzadeh et al. 2022). *DIAPH1* gene comprises 28 exons and is located on the long arm of chromosome 5 (5q31). Expression of the *DIAPH1* gene has been detected in human forebrain and neural progenitor cells. It has been shown that *DIAPH1* has a crucial role in brain development (Ercan‐Sencicek et al. 2015).

In the present study, we reported a novel pathogenic variant in *DIAPH1* gene in a 7‐year‐old boy with clinical manifestations of seizures, visual impairment, and mild mental retardation.

## 2 Materials and Methods

### 2.1 Compliance With Ethical Standards

The current study was approved by the Ethics Committee of the Isfahan University of Medical Sciences (IR.ARI.MUI.REC.1400.011). The research related to human use has complied with all the relevant national regulations, and institutional policies, following the tenets of the Helsinki Declaration, and has been approved by the author's institutional review board or equivalent committee.

### 2.2 DNA Extraction and Molecular Testing

Genomic DNA from the peripheral blood samples of the patient and his parents was extracted according to the salting‐out protocol. The quality of DNA extraction is checked using 1% agarose gel electrophoresis. The proband was tested for FMR1 CGG repeats to rule out Fragile X syndrome (FXS). It was done using the Deviner Fragile X (FMR1 Gene) Carrier Screen Kit (provided by KEYSAR Company, Tehran, Iran).

### 2.3 Exome Sequencing (ES)

Genomic DNA from the peripheral blood samples of the patient and his parents was extracted according to the salting‐out protocol. The patient‐extracted DNA was fragmented and enriched for ES using an Agilent SureSelect V7 kit. The libraries were sequenced to mean > 90× coverage on an Illumina Novaseq 6000 platform. The initial sequencing component of this test was performed by the Illumina genome sequencing service in Macrogen (Seoul, Korea).

### 2.4 Data Analysis

After sequencing, raw data were transformed into the FASTQ file. Aligning the sequencing results to the human reference genome (GRCh37/hg19) was performed using the Burrows–Wheeler–Aligner (BWA) program, and variant calls were made using the Genomic analyzer tool kit (GATK). Picard to mark duplicate reads was used. Gene annotation of the variants is performed using the Variant Effect Predictor (VEP) program. Variant filtering was performed based on homozygous missense, start codon change, splice site, nonsense, stop loss, and indel variants with minor allele frequency < 1% in databases, such as dbSNP version 147, 1000 genomes. Common variants are filtered based on the available information from databases (including HGMD, ClinVar, 1000 Genome, ExAC, LSDBs, dbSNP, and the local database, Iranome), published literature, clinical correlation, and their predicted function.

### 2.5 Bioinformatics Tools

We used the Genome Aggregation Database (gnomAD v4.0.0) for population allele frequency analysis. The potential pathogenicity of the variants was assessed using the following prediction tools: FATHMM & FATHMMMKL (Functional Analysis through Hidden Markov Models (v2.3), http://fathmm.biocompute.org.uk), LIST‐S2 (https://list‐s2.msl.ubc.ca/?session=28AB3E5B08FD16AF971162581885ACC2), M‐CAP (Mendelian Clinically Applicable Pathogenicity, http://bejerano.stanford.edu/mcap/), Mutation assessor (http://mutationassessor.org/), MutPred (http://mutpred.mutdb.org/), PROVEAN (PROVEAN scores (v1.1)), SIFT (Scale‐Invariant Feature Transform, https://sift.bii.a‐star.edu.sg) & SIFT4G, MutationTaster (https://www.mutationtaster.org/), BayesDel (addAF and noAF) (https://fenglab.chpc.utah.edu/BayesDel/), MetaLR (e!Ensembl https://useast.ensembl.org/index.html), MetaRNN (http://www.liulab.science/metarnn.html), REVEL (Rare Exome Variant Ensemble Learner,e!Ensembl https://useast.ensembl.org/index.html), and DEOGEN2 (http://deogen2.mutaframe.com/).

### 2.6 Sanger Sequencing Validation and Segregation Analysis

Finally, Sanger sequencing was done for validation of the detected variant. Specific primers were designed to amplify the target region of *DIAPH1* by polymerase chain reaction (PCR) (F: 5′‐TCCTAGCAAAGATGCTGGTTATG‐3′, R: 5′‐AGATAGGGTAGGGTAGCTGATG‐3′). Segregation analysis in the family of the patient was conducted to confirm the segregation of the detected variant with the phenotype.

## 3 Results

### 3.1 Case Presentation

**FIGURE 1:**
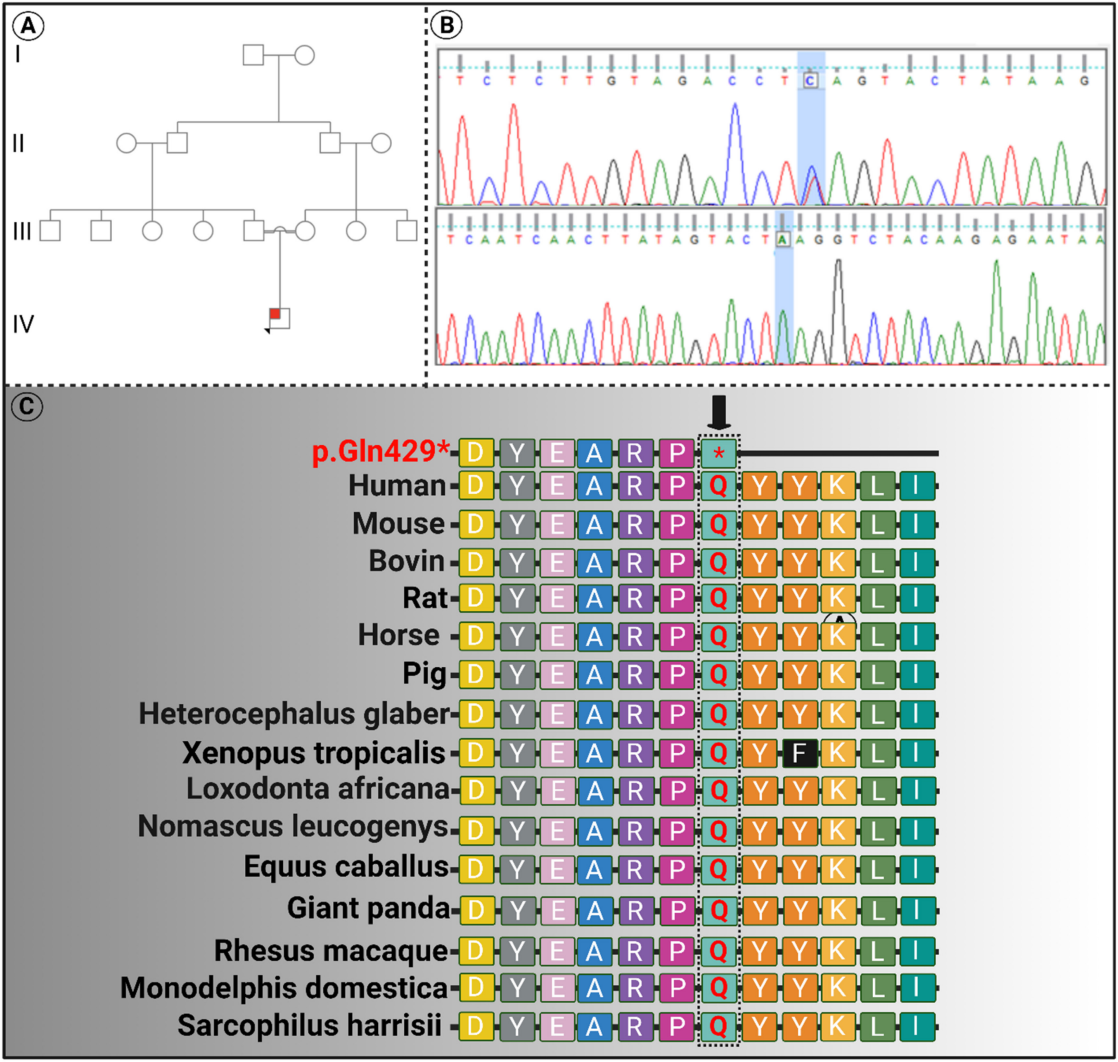
(A) Pedigree chart of the family. The black arrow in the pedigree specifies the proband. (B) Electropherogram of the family members; as shown, the proband was heterozygous for the c.1285C> T variant, but both unaffected parents harbored a wild‐type variant. (C) Conservation analysis showed that the *DIAPH1* variant occurred at highly conserved residues through evolution across several species. Black boxes indicate residues that differ from the normal human DIAPH1 protein sequence.

An Iranian 7‐year‐old boy was referred to our center with clinical manifestations of seizures, visual impairment, and mild mental retardation. A comprehensive genetic counseling was performed for the family and the family pedigree was drowned. The pedigree of the SCBMS families with an autosomal recessive pattern of inheritance is shown in Figure 1. Peripheral blood karyotype was performed for the patient, and also, the patient was evaluated for FXS. The results of both tests were normal. The pedigree of this family revealed that the patient was the consequence of a first‐cousin marriage. Based on the results of genetic counseling, an autosomal recessive disorder was suspected. So, ES was recommended for the patient.

### 3.2 Molecular and Bioinformatics Analyses

**FIGURE 2:**
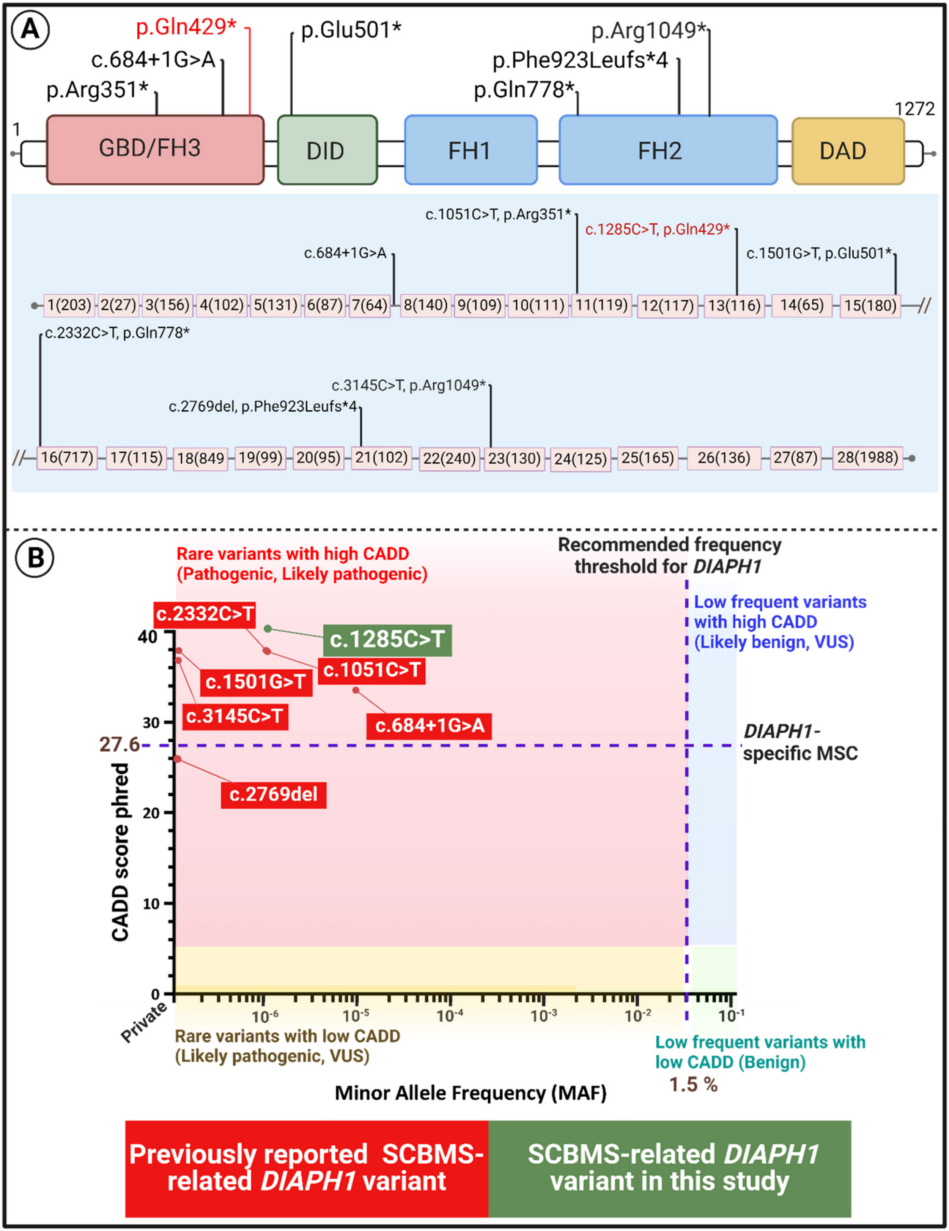
(A) Gene structure and the exon numbers with their sizes (bps) are indicated in parentheses. The introns are not drawn to scale. (B) A diagram depicting six previously reported pathogenic and likely pathogenic SCBMS‐related DIAPH1 variants. The *y*‐axis depicts the CADD scores of these variants. The mutation significance cutoff (MSC) of 27.6, which is specific for DIAPH1, is the maximum predicted CADD score tolerable by DOCK2 at a 99% significance level. The *x*‐axis depicts the allele frequency of these variants in different population databases, including the aggregated gnomAD database (https://gnomad.broadinstitute.org). Created with BioRender.com.

Analyzing the data obtained from ES identified a novel homozygous c.1285C> T (p.Gln429*) variant in Exon 13 of the *DIAPH1* gene in the patient. The nonsense c.1285C> T (p.Gln429*) variant in the *DIAPH1* gene causes termination of translation at amino acid 429 of 1272. Termination at amino acid 429 located at the first domain (DID) creates the truncated protein lacking 844 amino acids (the whole part of the CC, FH1, FH2, and DAD domains and 21 amino acids from the DID). Since the p.Gln429* variant affects a large portion of the protein, the pathogenic effect is predicted (Figure 2).

Analysis of the detected variant in the family using the Sanger sequencing method, confirmed the segregation of the detected variant with the phenotype (Figure 1). According to the ACMG guideline of variant classification (Richards et al. 2015), the identified variant was classified as “Pathogenic” because of having the following criteria for the pathogenicity: PVS1(null variant), PP3 (In silico predictions), PM2 (Extremely low frequency in gnomAD population databases), PP1 (cosegregation), and PP4 (patient's phenotype or family history is highly specific for a disease with a single genetic etiology).

The combined annotation‐dependent depletion (CADD) score was 40, well above the mutation significance cutoff (MSC) of 27.6 (Kircher et al. 2014; Itan et al. 2016; Zhang et al. 2018).

### 3.3 Literature Review and Genotype/Phenotype Correlation

We searched PubMed, Scopus, Web of Science (science and social science citation index), and Google Scholar for articles published before February 2024. Article selection criteria included: (i) case reports of *DIAPH1* biallelic variants; (ii) individuals who have both defined variants and clinical manifestations; and (iii) language was limited to English.

We reviewed the literature and found 20 patients from seven studies. Six different variants were identified. The most common variants include c.2769del, c.684 + 1G>A, and c.2332C>T which were present in 15 patients (Table 1). Among the 20 patients, the nonsense variant was the major type with a frequency of 11 (53% of patients).

**TABLE 1:** Bioinformatics software prediction of the adverse effects of the *DIAPH1* variant.

Genetic variant	MutationTaster	DANN	BayesDel	GenoCanyon	fitCons	EIGEN	FATHMM‐MKL	Pathogenicity line of evidence, based on ACMG
c.1285C>T	Deleterious	Deleterious	Deleterious	Deleterious	Deleterious	Pathogenic	Pathogenic	PVS1, PP3, PM2, PP1, PP4

The mean age of onset for *DIAPH1* biallelic variants in the group of 20 patients with a known age of onset was 2.3 months (SD = 1.6), with a median age of onset of 2.25 months. According to the literature, 65% of patients with SCBMS are alive (13 out of 20 determined cases).

Among the 21 patients carrying the *DIAPH1* biallelic variants (including the case reported in this study), 10 (50%) were male patients, and 10 (50%) were female patients (one gender was not reported) (Table 2). Seventy‐three percent (13 out of 18 determined cases) and 71% (12 out of 17 determined cases) of the patients indicated consanguinity and a positive family history of immunodeficiency, respectively (Table 3).

**TABLE 2:** Genotype of 21 patients with SCBMS‐related *DIAPH1* biallelic variants.

No.	Test of genetic diagnosis	gnomAD v4.0.0 (Aggregated)	cDNA variant	rs‐Number	Protein variant	Type of variant (coding impact)	Location	ACMG	CADD PHRED	Frequency (*n* = 21)	Percentage (%)	Ref.
1	ES	NA	c.2769del	rs863225242	p.Phe923Leufs*4	Del (Frameshift)	21	P	26	5	24%	Al‐Maawali et al. (2016); Kaustio et al. (2021)
2	ES	0.00003	c.684 + 1G>A	rs766545876	—	SNV (Splicing)	7	LP	34	5	24%	Kaustio et al. (2021)
3	ES	0.000004	c.2332C>T	rs730882242	p.Gln778*	SNV (Nonsense)	16	P	38	5	24%	Ercan‐Sencicek et al. (2015)
4	ES	NA	c.3145C>T	rs863225243	p.Arg1049*	SNV (Nonsense)	23	P	37	3	14%	Al‐Maawali et al. (2016); Esmaeilzadeh et al. (2022); Yavarna et al. (2015)
5	ES	0.000002	c.1051C>T	rs755174598	p.Arg351*	SNV (Nonsense)	11	P	38	1	4.5%	Acer et al. (2017)
6	ES	0	c.1501G>T	—	p.Glu501*	SNV (Nonsense)	15	LP	38	1	4.5%	Dhami and Izadi (2021)
7	ES	0.000001	c.1285C>T	—	p.Gln429*	SNV (Nonsense)	13	LP	40	1	4.5	Current study

**TABLE 3:** Phenotype of 21 patients with SCBMS‐related *DIAPH1* biallelic variants.

P.	Sex	Age (Y) /age of onset (M)	Ethnicity	Consanguinity/ family history	Seizures	Cortical blindness	Microcephaly	DD*	Poor speech	Short stature	Hypotonia	Poor growth	Brain imaging	Functioning level	Recurrent infections	Current status	Ref.
1	M	2.75/0.5	United Arab Emirates	+/−	+	+	+	+	+	+	−	+	Abnormal	Could walk, feed self with hands but no utensils, follow simple commands	+	Alive	Al‐Maawali et al. (2016)
2	F	13/4	Omani	+/+	+	+	+	+	−	+	−	+	ND	Could walk, cognition like 1 y old, some head banging	+	Dead (Pneumonia)	Al‐Maawali et al. (2016)
3	F	0.75/3	Omani	+/+	+	+	+	+	−	−	+	+	ND	Could sit, low tone, global motor delays	ND	Dead (Seizures)	Al‐Maawali et al. (2016)
4	M	1.75/0.26	Omani	+/+	+	+	+	+	−	ND	+	+	Abnormal (corpus callosum)	Low tone	ND	Alive	Al‐Maawali et al. (2016)
5	M	4/0	ND	ND/ND	+	+	+	+	−	+	−	+	Thin corpus calusom splenium suggestive of mid‐central cortical atrophy	ND	+	Alive	Esmaeilzadeh et al. (2022)
6	ND	ND	ND	ND/ND	+	−	+	+	ND	ND	ND	ND	ND	ND	−	ND	Yavarna et al. (2015)
7	F	15/3	Saudi Arabia	+/+	+	+	+	+	+	−	−	−	−	Poor adaptive skills, Could walk, sit, and explore objects with her hands	−	Alive	Ercan‐Sencicek et al. (2015)
8	F	14/3	Saudi Arabia	+/+	+	+	+	+	+	+	−	+	Prominent cortical sulci and gyri of occipital lobes	Could walk, sit, and explore objects with hands	+	Dead (Pneumonia)	Ercan‐Sencicek et al. (2015)
9	F	6/3	Saudi Arabia	+/+	+	+	+	+	+	+	−	+	Normal Axial T1,2	Could walk, sit, and explore objects with hands	−	Alive	Ercan‐Sencicek et al. (2015)
10	M	2/3	Saudi Arabia	+/+	+	+	+	+	+	+	−	+	Temporal pole atrophy with normal cortical thickness and increased CSF space in the middle cranial fossa anterior to the temporal pole, dilatation of the lateral ventricles, small splenium of the corpus callosum	Could walk, sit, and explore objects with hands	−	Alive	Ercan‐Sencicek et al. (2015)
11	M	0.25/3	Saudi Arabia	+/+	+	+	+	+	+	+	−	+	NA	ND	−	Alive	Ercan‐Sencicek et al. (2015)
12	M	ND/2	Finland	−/+	+	+	+	+	ND	ND	ND	ND	Abnormal signal intensities in occipital white matters; thin chiasm and optic nerves	ND	+	Dead (B‐cell lymphoma)	Kaustio et al. (2021)
13	M	ND/2	Finland	−/+	+	+	+	+	ND	ND	ND	ND	Abnormal signal intensities in occipital white matters; thin chiasm and optic nerves	ND	+	Dead (Status epilepticus)	Kaustio et al. (2021)
14	M	7/2.5	Finland	−/−	+	+	+	+	ND	ND	ND	ND	Abnormal signal intensities in occipital white matters; thin chiasm and optic nerves	ND	+	Alive	Kaustio et al. (2021)
15	F	3/0.06	Finland	−/−	+	+	+	+	ND	ND	ND	ND	−	ND	+	Alive	Kaustio et al. (2021)
16	F	ND/1	Finland	−/−	+	+	+	+	ND	ND	ND	ND	Occipital atrophy, encephalitis	ND	+	Dead (Pneumonia, encephalitis)	Kaustio et al. (2021)
17	F	ND/2	Oman	+/+	+	+	+	+	ND	ND	ND	ND	Brain atrophy	ND	+	Dead(B‐cell lymphoma)	Kaustio et al. (2021)
18	M	3/0.5	Oman	+/+	+	+	+	+	ND	ND	ND	ND	Partial agenesis of corpus callosum, occipital atrophy	ND	+	Alive	Kaustio et al. (2021)
19	F	29/5	Turkey	ND	+	+	+	+	ND	+	+	+	Bilateral parietooccipital parasagittal area volume loss and T2 signal increase is available white matter; bilateral optic discs atrophy	ND	−	Alive	Acer et al. (2017)
20	F	2/6	ND	+/ND	+	+	+	+	ND	+	ND	+	ND	ND	+	Alive	Dhami and Izadi (2021)
21	M	7/2	Iranian	+/−	+	+	+	+	+	+	ND	+	ND	Poor adaptive skills	−	Alive	Current study

* DD = Developmental Delay.

**FIGURE 3:**
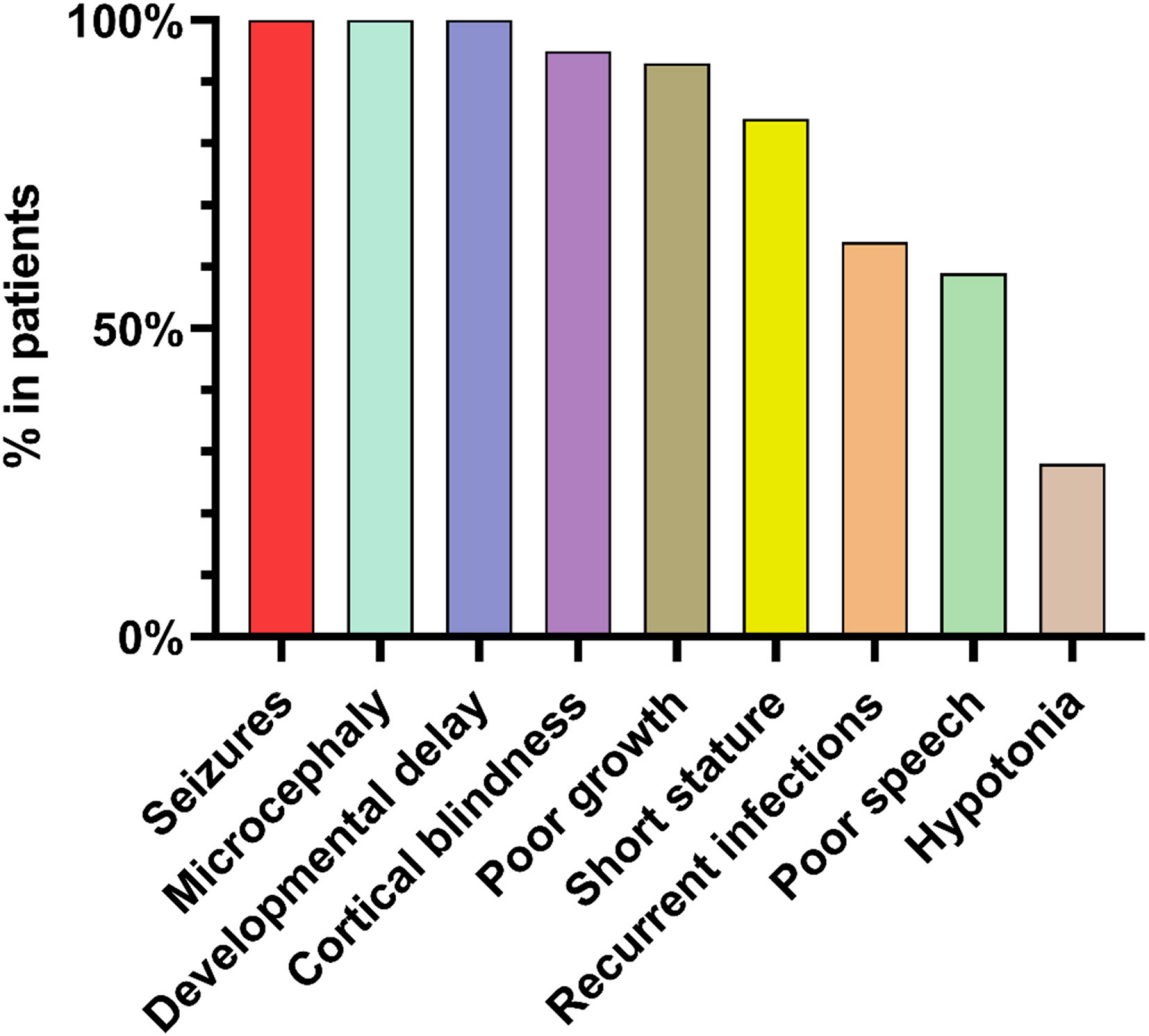
The most common clinical presentation in patients with SCBMS‐related DIAPH1 biallelic variants.

Of the patients whose phenotype was reported, three phenotypes were present in 100% of the patients (seizures, developmental delay, and microcephaly). Other most common phenotypes include cortical blindness (20 out of 21, 95%), poor growth (12 out of 13, 93%), short stature (10 out of 12, 84%), recurrent infections (12 out of 19, 64%), and poor speech (7 out of 12, 64%) (Figure 3).

## 4 Discussion

ES is now extremely strong and affordable. It is becoming more labor‐cost‐efficient to prefer ES to other means of screening, particularly in rare diseases with a recessive pattern. ES has made microcephaly, one of the genetically most heterogeneous conditions, accessible to routine genetic testing. With its increasing diagnostic application, the individual contributions of pathogenic variants in the known genes to microcephaly are becoming clearer. Furthermore, genotype–phenotype correlations can be verified through patients with pathogenic variants in the same gene. Using ES and subsequent Sanger validation monitored by cosegregation analysis of the known *DIAPH1* gene in an SCBMS patient showed a novel stop gain pathogenic variant that segregated with the SCBMS phenotype, and we gave a definite molecular diagnosis for her.

mDia1 is a protein that, in humans, is encoded by the *DIAPH1* gene and has an essential role in the regulation of cell morphology and cytoskeletal organization (Shinohara et al. 2012; Tominaga et al. 2000). It has been demonstrated that disease‐causing variants in the *DIAPH1* gene have a close association with two diseases. The first homozygous pathogenic variant in the *DIAPH1* gene that causes microcephaly has been detected in the Saudi Arabian population (Ercan‐Sencicek et al. 2015). Since then, variants in this gene have been reported in various neurodevelopmental disorders. Damaging variants in this gene were detected in the finish population and Omani patients with microcephaly syndrome, immunodeficiency, and mitochondrial dysfunction (Ganaha et al. 2017). A pathogenic variant in the *DIAPH1* gene was identified in a Japanese family with progressive hearing loss and macrothrombocytopenia (Kaustio et al. 2021). A homozygous autosomal recessive pathogenic variant in *DIAPH1* has been detected in an Iranian boy with microcephaly syndrome and immunodeficiency (Esmaeilzadeh et al. 2022).

*DIAPH1* is expressed in the human medial temporal cortex, particularly in myeloid cells (Derk et al. 2018). During embryogenesis, expression of the *DIAPH1* gene has been detected in the ventricular and subventricular zone progenitor cells of the forebrain and the brainstem. During postnatal development, *DIAPH1* is expressed in the cerebral cortex, basal ganglia, hippocampus, thalamus, and external granular layer of the cerebellum. Expression of *DIAPH1* is associated with the centrosomes and mitotic spindle (Ercan‐Sencicek et al. 2015).

The mDia protein acts downstream of Rho GTPases and upstream of PFN (profiling) protein. Profilins are small actin‐binding proteins that play an important role in regulating actin polymerization. Profilins are expressed in the central nervous system. Neurological diseases such as Huntington's disease (HD), FXS, and spinal muscular atrophy (SMA) are associated with Profilins (Esmaeilzadeh et al. 2022; Murk, Ornaghi, and Schiweck 2021). Altogether, data obtained from expression patterns of *DIAPH1* and pathogenic variants in this gene in two diseases show that *DIAPH1* has a crucial role in brain development, and a defect in this gene may cause a neurological disorder. In this study, a homozygous c.1285C>T pathogenic variant was detected in a 7‐year‐old boy with clinical manifestations of seizures, visual impairment, and mild mental retardation.

Based on the pathogenic variant location in the *DIAPH1* gene and encoded protein, the damaging effect of this variant is considered. In silico analysis with prediction software tools also identified this variant as a probable source of damage. Analysis of the detected variant in the family confirmed our finding and revealed that the patient's parents are in the heterozygote state. So, due to the consanguineous marriage, it seems that the parents have inherited this variant from a common ancestor. Identification of the genetic cause of the disease for this family allowed the parents to evaluate the risk of having an affected child in future pregnancies and arrange appropriate management. Although the detailed functional mechanism of this nonsense variant needs more investigation, the progressive identification of genetic defects associated with these complications will eventually reveal the underlying pathological mechanisms and help develop more effective treatment strategies for the disease.

In conclusion, we report a male individual with a homozygous nonsense variant in the *DIAPH1* gene. This nonsense variant truncates the GBD/FH3 domain and removes over half of the DIAPH1 protein. Besides, by investigating all of the reported molecularly confirmed cases, we provided a comprehensive picture of clinical symptoms of patients with SCBMS‐related *DIAPH1* biallelic variants.

## Author Contributions

Emran Esmaeilzadeh and Sajjad Biglari were involved in the recruitment of patient and family members, mutation screening and segregation analysis in families by Sanger sequencing, and writing the first draft of the manuscript; Meysam Mosallaei, Hassan Vahidnezhad, and Hamid Reza Khorram Khorshid were involved in writing the first draft of the manuscript and manuscript revision; Mohammad Amin Tabatabaiefar was involved in the conceptualization and supervision of the research, review and editing the first draft of the manuscript. All of the authors read and approved the final manuscript to be published and agreed to be responsible for the accuracy of the data and details.

## Conflicts of Interest

The authors declare no conflicts of interest.

## Data Availability

The data that support the findings of this study are available from the corresponding author upon reasonable request.
